# BRCA1 and BRCA2 Germline Mutations in Malaysian Women with Early-Onset Breast Cancer without a Family History

**DOI:** 10.1371/journal.pone.0002024

**Published:** 2008-04-23

**Authors:** Gaik Theng Toh, Peter Kang, Sharlene S. W. Lee, Daphne Shin-Chi Lee, Sheau Yee Lee, Suhaida Selamat, Nur Aishah Mohd Taib, Sook-Yee Yoon, Cheng Har Yip, Soo-Hwang Teo

**Affiliations:** 1 Cancer Research Initiatives Foundation (CARIF), Outpatient Centre, Subang Jaya Medical Centre, Selangor, Malaysia; 2 Department of Surgery, University Malaya Medical Centre, Kuala Lumpur, Malaysia; University of Sydney, Australia

## Abstract

**Background:**

In Asia, breast cancer is characterised by an early age of onset: In Malaysia, approximately 50% of cases occur in women under the age of 50 years. A proportion of these cases may be attributable, at least in part, to genetic components, but to date, the contribution of genetic components to breast cancer in many of Malaysia's ethnic groups has not been well-characterised.

**Methodology:**

Given that hereditary breast carcinoma is primarily due to germline mutations in one of two breast cancer susceptibility genes, *BRCA1* and *BRCA2*, we have characterised the spectrum of *BRCA* mutations in a cohort of 37 individuals with early-onset disease (≤40 years) and no reported family history. Mutational analysis of *BRCA1* and *BRCA2* was conducted by full sequencing of all exons and intron-exon junctions.

**Conclusions:**

Here, we report a total of 14 *BRCA1* and 17 *BRCA2* sequence alterations, of which eight are novel (3 *BRCA1* and 5 *BRCA2*). One deleterious *BRCA1* mutation and 2 deleterious *BRCA2* mutations, all of which are novel mutations, were identified in 3 of 37 individuals. This represents a prevalence of 2.7% and 5.4% respectively, which is consistent with other studies in other Asian ethnic groups (4–9%).

## Introduction

Carcinoma of the breast is the most prevalent female malignancy worldwide [1]. However, there is considerable geographical variation in disease rates whereby the highest age-standardised incidence rates (ASR) are found in developed regions in the West such as North America and Europe [1], [2]. Incidence rates in developing areas and countries of the Far East, albeit on the rise, are substantially lower.

In Malaysia, breast cancer constituted 31% of all newly diagnosed female cancer cases in 2003, making it the most common cancer amongst Malaysian women across all major ethnic groups [3]. The overall incidence rate in 2003 (ASR of 46.2 per 100,000) is comparable to that of Singapore and the Philippines, but are relatively higher than those reported in China, Japan, India and Thailand [2]. It is also noteworthy that approximately half of the cases reported in Malaysia occurred in women under the age of 50 years. The apparent lower mean age of onset compared to the Western population has also been reported in other Asian countries [1]. One possible explanation is the “Westernization” of these populations [4], i.e. delayed childbearing, lower parity, reduced breast-feeding, decreased exercise and dietary changes, has led to a ‘cohort effect’ where the younger cohort have increased breast cancer risk compared to their mothers and grandmothers, thus giving rise to an apparent lower mean age of onset. However, given that an individual with breast cancer is more likely to carry a susceptibility gene mutation the younger she is at the time of diagnosis [5]–[7], an alternative possibility is that the allelic frequency of high penetrance genes in the Asian population may be higher than that in Caucasian populations.

Hereditary breast carcinomas are predominantly a consequence of loss-of-function germline mutations in one of two major breast cancer susceptibility genes, *BRCA1* and *BRCA2* [8]–[10]. Epidemiological studies suggest that deleterious mutations in either of these genes confer a significant increase in lifetime risk of developing breast and, to a lesser extent, ovarian cancers [11]–[14]. It is argued that this estimated risk is nonetheless imprecise and may be further modified by other genetic and non-genetic factors [15].

Our current understanding of *BRCA* mutations and their contribution to hereditary breast cancer has arisen primarily from studies conducted in Caucasian women. A number of studies which have been carried out in several Asian countries, however, indicate that *BRCA* mutations are equally prevalent or may possibly be higher within the Asian population [16]. The majority of studies to date, particularly those in South East Asia, have focused primarily on *BRCA1*, with few comprehensive reports on the *BRCA2* gene. In women with early-onset breast carcinoma unselected for family history, the prevalence of *BRCA1* mutations is estimated to be between four and nine percent [16]–[23]. Founder mutations that are unique to Asians have also been described [16]. Despite this emerging knowledge, little is known about the spectrum and frequency of *BRCA* mutations and their involvement in breast cancer incidence within the ethnically diverse Malaysian population of Malays, Chinese & Indians [24]. This report describes sequence variants of *BRCA1* and *BRCA2* that were identified in a hospital-based cohort of Malaysian women with early onset breast cancer (≤40 years) with no reported family history of breast and/or ovarian cancers.

## Results

The Malaysian population is characterised by three major ethnic groups: the Malays (62%), Chinese (27%) and Indians (9%). Of the 3,738 new cases reported in 2003, 43.6% of patients were Chinese, while 39.7% were Malay and 11% were of Indian origin. Notably, the age-standardised incidence is higher among the Chinese and Indians in comparison to the Malays [3].

Patients for this study were recruited from one major breast cancer referral hospital in Malaysia, the University Malaya Medical Centre (UMMC), Kuala Lumpur. Due to its demographics, a larger proportion of patients i.e. 59.4% treated at this hospital were ethnically Chinese, compared to 25.8% Malays and 13.4% Indians. However, comparisons between the hospital and population data set show that collectively, breast cancer cases managed by UMMC over a ten-year period from 1996–2005 essentially mirror those that were reported to the national registry in 2003. Firstly, approximately 50% of cases in the population and those seen at the hospital occurred in women under the age of 50 years, 15% of whom were 40 years and below. Secondly, the most prevalent age group in both the population and hospital-based series was 40–49 years, whereby an average of 34% of all cases lies within this age bracket. Thirdly, discernable differences that are observed between the three ethnic groups are found in both the population and hospital data sets. In women below the age of 50 years, breast cancer incidence increased with age in all three ethnic groups. However, after the age of 50, incidence decreased in the Chinese and Malays but continued to rise within the Indian sub-population. A similar trend has been noted in an extensive study of trends of breast cancer incidence in women in Singapore, a country that is characterised by the same major ethnic groups, albeit in significantly different proportions [25]. Finally, it is clear that in both the population- and hospital-based series, disease onset in Malay women occur at a younger age in comparison to the Chinese and Indians. Taken together, this suggests that the hospital-based cohort is largely reflective of breast cancer cases within the Malaysian population.

A total of 37 proband patients diagnosed with early-onset breast cancer (≤40 years) with a mean age of 33.7 years and no known family history of the disease were selected for this study. The ethnicity of this study group reflected the ethnicity of the hospital-based sample collection i.e. 21 Chinese (56.8%), 11 Malays (29.7%), 4 Indians (10.8%) and 1 individual of mixed race (2.7%). Within this cohort, we identified a total of 14 *BRCA1* and 17 *BRCA2* sequence variants, of which ten are novel (summarised in Tables 1 & 2).

**Table 1:** Sequence variants identified in the *BRCA1* gene.

	Clinical relevance	Type	Exon	Nucleotide change (HGVS)	Nucleotide change	AA Change	Novel/Reported (BIC)	Frequency, n = 37	Ethnicity
1	Deleterious	IVS	3	IVS3+2delT	IVS3+2delT	NA	Novel	1/37, 2.7%	Chinese
2	VUS	MS	11	c.2286 A>T	2405 A>T	R762S	Reported, [26]	1/37, 2.7%	Malay
3	VUS	MS	11	c.2566 T>C	2685 T>C	Y856H	Reported	1/37, 2.7%	Chinese
4	Benign*	Syn	11	c.1873 C>T	1992 C>T	No change (L625L)	Novel	1/37, 2.7%	Chinese
5	Benign*	Syn	11	c.2931 A>G	3050 A>G	No change (P977P)	Novel	1/37, 2.7%	Chinese
6	Polymorphism	Syn	3	c.114 G>A	233 G>A	No change (K38K)	Reported	1/37, 2.7%	Chinese
7	Polymorphism	MS	11	c.2077 G>A	2196 G>A	D693N	Reported	1/37, 2.7%	Chinese
8	Polymorphism	Syn	11	c.2082 C>T	2201 C>T	No change (S694S)	Reported	28/37, 75.7%	Malay (10/11), Chinese (15/21), Indian (3/4)
9	Polymorphism	Syn	11	c.2311 T>C	2430 T>C	No change (L771L)	Reported	28/37, 75.7%	Malay (10/11), Chinese (15/21), Indian (3/4)
10	Polymorphism	MS	11	c.2612 C>T	2731 C>T	P871L	Reported	28/37, 75.7%	Malay (10/11), Chinese (15/21), Indian (3/4)
11	Polymorphism	MS	11	c.3113 A>G	3232 A>G	E1038G	Reported	28/37, 75.7%	Malay (10/11), Chinese (15/21), Indian (3/4)
12	Polymorphism	MS	11	c.3548 A>G	3667 A>G	K1183R	Reported	28/37, 75.7%	Malay (10/11), Chinese (15/21), Indian (3/4)
13	Polymorphism	Syn	13	c.4308 T>C	4427 T>C	No change (S1436S)	Reported	28/37, 75.7%	Malay (10/11), Chinese (15/21), Indian (3/4)
14	Polymorphism	MS	16	c.4837 A>G	4956 A>G	S1613G	Reported	28/37, 75.7%	Malay (10/11), Chinese (15/21), Indian (3/4)

MS, missense; Syn, synonymous; FS, frameshift; VUS, variant of uncertain significance; BIC, Breast Information Core

**Table 2:** Sequence variants identified in the *BRCA2* gene.

No	Clinical relevance	Type	Exon	Nucleotide change (HGVS)	Nucleotide change	AA Change	Novel/Reported (BIC)	Frequency, n = 37	Ethnicity
1	Deleterious	FS	11	c.2634 delCT	2862 delCT	STOP 879	Novel	1/37, 2.7%	Chinese
2	Deleterious	FS	11	c.6673 delA	6901 delA	STOP 2228	Novel	1/37, 2.7%	Chinese
3	VUS	MS	10	c.943 T>A	1171 T>A	C315S	Reported	1/37, 2.7%	Chinese
4	VUS	MS	11	c.3445 A>G	3673 A>G	M1149V	Reported	1/37, 2.7%	Malay
5	VUS	MS	11	c.5167 A>C	5395 A>C	T1723P	Novel	1/37, 2.7%	Malay
6	Benign*	Syn	10	c.1275 A>G	1503 A>G	No change (E425E)	Reported, [29]	1/37, 2.7%	Chinese
7	Benign*	Syn	11	c.4578 A>G	4806 A>G	No change (T1526T)	Novel	1/37, 2.7%	Mixed
8	Benign*	Syn	17	c.7941 A>C	8169 A>C	No change (L2647L)	Novel	1/37, 2.7%	Malay
9	Polymorphism	MS	10	c.865 A>C	1093 A>C	N289H	Reported	3/37, 8.1%	Malay (1/11), Chinese (2/21)
10	Polymorphism	MS	10	c.1114 C>A	1342 C>A	H372N	Reported	19/37, 51.4%	Malay (5/11), Chinese (12/21), Indian (1/4), Mixed (1/1)
11	Polymorphism	Syn	10	c.1365 A>G	1593 A>G	No change (S455S)	Reported	3/37, 8.1%	Malay (1/11), Chinese (2/21)
12	Polymorphism	Syn	11	c.2229 T>C	2457 T>C	No change (H743H)	Reported	3/37, 8.1%	Malay (1/11), Chinese (2/21)
13	Polymorphism	MS	11	c.2971 A>G	3199 A>G	N991D	Reported	3/37, 8.1%	Malay (1/11), Chinese (2/21)
14	Polymorphism	Syn	11	c.3396 A>G	3642 A>G	No change (K1132K)	Reported	22/37, 59.5%	Malay (4/11), Chinese (16/21), Indian (1/4), Mixed (1/1)
15	Polymorphism	Syn	11	c.3807 T>C	4035 T>C	No change (V1269V)	Reported	8/37, 21.6%	Malay (3/11), Chinese (5/21)
16	Polymorphism	MS	11	c.5312 G>A	5540 G>A	G1771D	Reported	1/37, 2.7%	Chinese
17	Polymorphism	Syn	14	c.7242 A>G	7470 A>G	No change (S2414S)	Reported	22/37, 59.5%	Malay (5/11), Chinese (16/21), Mixed (1/1)

* These sequence alterations are likely to be polymorphisms. They have been classified as benign as allelic frequency data from population studies are not currently available. MS, missense; Syn, synonymous; FS, frameshift; VUS, variant of uncertain significance; BIC, Breast Information Core

### *BRCA1* Sequence Variants

Fourteen *BRCA1* sequence variants, including three novel changes, were identified in this study population (Table 1). Only one clearly disease-associated mutation was found in this cohort. The splice site mutation (IVS3+2delT), which occurs within the proximal splice site of exon 3, was detected in one patient of Chinese descent.

Two *BRCA1* missense variants of uncertain significance were identified in two out of 37 proband patients (5.4%). One variant at position 2405 (A>T) in exon 11 that led to a substitution of Ser for Arg residue at position 762 (R762S) was detected in an individual of Malay descent. Although its clinical significance is presently unknown, this substitution of this invariant Arg [27], which occurs outside any known functional domain, is unlikely to contribute to increased risk to breast cancer because it has been reported to occur in a cohort of cancer-free individuals [26].

The second *BRCA1* variant of uncertain significance was identified in a Chinese patient. It involved an alteration at position 2685 (T>C) that resulted in a Tyr to His substitution at codon 856 (Y856H) in exon 11. This variant has been reported previously occurring at similar frequencies in breast cancer and cancer-free individuals of Chinese descent in Hong Kong [18] and Shanghai [26], suggesting that this variant is neutral or of little pathological significance.

Eleven additional *BRCA1* sequence variants were identified in this study group (Table 1). These include two novel changes in exon 11 that were detected in two Chinese patients. These variants are considered benign and may be polymorphisms as the single base alterations do not lead to amino acid substitutions (L625L and P977P) and are unlikely to be potential splice site variants. A further nine variants that were identified have been reported previously in BIC and are classified as neutral polymorphisms. Two alterations, a synonymous alteration in exon 3 (K38K) and a missense substitution in exon 11 leading to an Asp for Asn substitution at codon 693 (D693N), are rare and were only identified once each (1/37, 2.7%) in two Chinese patients. In contrast, seven other single nucleotide polymorphisms were found in 75.7% (28/37) of individuals across all three ethnic groups in this patient population. These correspond to three silent substitutions in exon 11 (S694S and L771L) and exon 13 (S1436S), and four missense changes in exon 11 (P871L, E1038G, K1183R) and exon 16 (S1613G). This block of seven common polymorphisms always occurred concurrently i.e. a patient that carries one variant was always found to carry the other six variants. Similarly, five of these polymorphic sites in exon 11 have been noted to co-occur in other Asian [18], [22], as well as Caucasian populations [8], [28].

### *BRCA2* Sequence Variants

A total of 17 *BRCA2* sequence variants, five of which have not been previously reported, were identified in this patient population (Table 2). These include two distinct frameshift mutations that are predicted to encode truncated proteins and three missense variants of uncertain clinical significance. The two deleterious frameshift mutations were detected in two patients (5.4%) of Chinese descent. The first is a 2-bp deletion at nucleotide 2862 (2862 delCT) while the second involves the deletion of adenosine at position 6901 (6901 delA). These two alterations are predicted to result in premature STOP codons at positions 879 (STOP 879) and 2228 (STOP 2228), respectively.

The three *BRCA2* variants of uncertain significance identified in this study correspond to substitutions in exons 10 and 11 (Table 2) and none are likely to be clinically relevant. One is a novel variant which encodes a Thr to Pro substitution at codon 1723. This variant was detected in an index case who also carries the novel *BRCA1* R762S variant of uncertain significance. Based on multiple sequence alignment analysis, this Thr residue is not highly evolutionarily conserved, with alternative amino acids being found in dog, mouse and chick. Furthermore, the pairwise GMS of 38 for a Thr to Pro change is relatively low. Taken together, this variant is not likely to be disease causative. Two other missense variants of uncertain significance, C315S and M1149V were detected in two patients of Chinese and Malay descent, respectively. These two variants have been reported in BIC, primarily in individuals of Asian origin. Based on multiple sequence alignment analysis, Grantham score (GMS = 112) and the SIFT (Sorting Intolerant From Tolerant [30]) prediction programme, the C315S missense mutation is not likely to affect protein function. In addition, the observations of a study conducted on Chinese women from Shanghai also argue against the deleterious nature of this mutation [26]. This sequence variant was identified in women with benign breast disease as well as individuals within the control cohort. Likewise, the M1149V variant is predicted to be a neutral alteration that has little clinical significance. This is because a Met to Val alteration is a conservative substitution, as indicated by a relatively low Grantham chemical difference score of 21, and occurs at a residue that is not evolutionarily conserved.

In addition to the two novel frameshift mutations and an unreported variant of uncertain significance, we identified three distinct synonymous mutations, of which two are novel, within this patient population (Table 2). These single nucleotide changes in exons 10 (E425E), 11 (T1526T) and 17 (L2647L) were identified once (1/37, 2.7%) in three patients and are unlikely to be potential splice site variants. Although these are most likely polymorphisms, they have been classified as clinically benign synonymous sequence variants because allelic frequency data from population studies are currently not available.

Nine previously reported common *BRCA2* polymorphisms were also identified in this study group. These polymorphisms corresponded to sequence alterations in exons 10, 11 and 14. Four sequence variants involved changes that resulted in amino acid substitutions (N289H, H372N, N991D and G1771D) while five were synonymous changes (S455S, H743H, K1132K, V1269V and S2414S) that did not affect the protein sequence of *BRCA2*. These nine polymorphic sites were found to occur at varying frequencies. One variant, G1771D, was identified in one of 37 patients (2.7%). Four of these, namely N289H and S455S in exon 10 and H743H and N991D in exon 11, occurred concurrently in three patients (3/37, 8.1%). The V1269V alteration in exon 11 was detected in 21.6% (8/37) of the study group while the three remaining polymorphisms, H372N (exon 10), K1132K (exon 11) and S2414S (exon 14), occurred at relatively high frequencies. They were detected in more than 50% of the cohort, with 28 patients carrying at least one of the three variants. Of these, 12 patients (12/28, 42.9%) were found to carry all three polymorphisms.

### Conclusion

Mutational analysis by direct sequencing of the entire coding region of the *BRCA1* and *BRCA2* genes on 37 breast cancer patients aged 40 years and under, with no reported family history of breast and/or ovarian cancers, identified a total of 14 *BRCA1* and 17 *BRCA2* sequence variants. These include ten novel mutations (4 *BRCA1* and 6 *BRCA2*) that have not been previously reported. One pathogenic *BRCA1* mutation and two novel pathogenic *BRCA2* mutations were detected in two of 37 index cases. This represents a prevalence of 2.7% and 5.4% respectively.

To date, only one other mutational analysis has been conducted on Malaysian breast cancer patients [24]. This study focused exclusively on the *BRCA1* gene. Clearly, there remain gaps in our understanding of how germline mutations in *BRCA* genes contribute towards breast cancer incidence in Malaysia, but given the relatively low yield of deleterious mutations in BRCA1 and BRCA2 despite a thorough examination of both genes, it might be concluded that germline mutations at these sites are unlikely to contribute to the observed lower age of onset of breast cancer compared to Western series.

## Materials and Methods

A total of 619 pathologically confirmed breast cancer patients were recruited from the University Malaya Medical Centre (UMMC) in Kuala Lumpur, Malaysia between January 2003 and December 2006. Family histories were obtained by interviewing patients. From these, 37 individuals with early onset disease (≤40 years) without any first and/or second degree relatives affected with breast and/or ovarian cancers were selected for this study cohort. Prior approval for this study was obtained from the University Malaya Medical Centre ethics committee, and written informed consent was obtained from all participating subjects.

DNA from peripheral blood was extracted using standard techniques and the entire coding sequence of *BRCA1* and *BRCA2* and splice sites (20 bp upstream and 10 bp downstream) were analysed for sequence variants by PCR and direct nucleotide sequencing. Samples were subjected to electrophoresis using MegaBACE™ 500 (Amersham Biosciences, Germany) and sequencing traces were analysed using the Mutation Surveyor v2.61 (Softgenetics Inc., USA) programme.

## References

[1] Ferlay J, Bray F, Pisani P, Parkin DM (2004). GLOBOCAN 2002: Cancer Incidence, Mortality and Prevalence Worldwide..

[2] Parkin DM (2004). International Variation.. Oncogene.

[3] Lim GCC, Halimah Y (2004). Second Report of the National Cancer Registry. Cancer Incidence in Malaysia 2003..

[4] Porter P (2008). “Westernizing” Women's Risk? Breast cancer in lower-income countries.. N Engl J Med.

[5] Claus EB, Schildkraut JM, Thompson WD, Risch NJ (1996). The genetic attributable risk of breast and ovarian cancer.. Cancer.

[6] Langston AA, Malone KE, Thompson JD, Daling JR, Ostrander EA (1996). *BRCA1* mutations in a population-based sample of young women with breast cancer.. N. Engl. J. Med..

[7] Fitzgerald MG, MacDonald DJ, Krainer M, Hoover I, O'Neil E (1996). Germ-line *BRCA1* mutation in Jewish and non-Jewish women with early-onset breast cancer.. N. Engl. J. Med..

[8] Miki Y, Swensen J, Shattuck-Eidens D, Futreal PA, Harshman K (1994). A strong candidate for the breast and ovarian cancer susceptibility gene *BRCA1*.. Science.

[9] Wooster R, Neuhausen SL, Mangion J, Quirk Y, Ford D (1994). Localisation of a breast cancer susceptibility gene, *BRCA2*, to chromosome 13q12-13.. Science.

[10] Wooster R, Bignell G, Lancaster J, Swift S, Seal S (1995). Identification of the breast cancer susceptibility gene *BRCA2*.. Nature.

[11] Ford D, Easton DF, Bishop DT, Narod SA, Goldgar DE (1994). Risks of cancer in *BRCA1* mutation carriers. Breast Cancer Linkage Consortium.. Lancet.

[12] Hopper JL, Southey MC, Dite GS, Jolley DJ, Giles GG (1999). Population based estimate of the average age-specific cumulative risk of breast cancer for a defined set of protein-truncating mutations in *BRCA1* and *BRCA2*. Australian Breast Cancer Family Study.. Cancer Epidemiol. Biomarkers Prev..

[13] Antoniou A, Pharoah PD, Narod S, Risch HA, Eyfjord JE (2003). Average risks of breast and ovarian cancer associated with *BRCA1* or *BRCA2* mutations detected in case series unselected for family history: a combined analysis of 22 studies.. Am J Hum Genet..

[14] Wooster R, Weber BL (2003). Genomic medicine: breast and ovarian cancer.. N. Engl. J. Med..

[15] Narod SA (2006). Modifiers of risk of hereditary breast cancer.. Oncogene.

[16] Leide A, Narod SA (2002). Hereditary breast and ovarian cancer in Asia: Genetic Epidemiology of *BRCA1* and *BRCA2*.. Hum. Mutat..

[17] Katagiri T, Emi M, Ito I, Kobayashi K, Yoshimoto M (1996). Mutations in the *BRCA1* gene in Japanese breast cancer patients.. Hum. Mutat..

[18] Tang NL, Pang CP, Yeo W, Choy KW, Lam PK (1999). Prevalence of mutations in the *BRCA1* gene among Chinese patients with breast cancer.. J. Natl. Canc. Inst..

[19] Ho GH, Phang BH, Ng IS, Law HY, Soo KC, Ng EH (2000). Novel germline *BRCA1* mutations detected in women in Singapore who developed breast carcinoma before the age of 36 years.. Cancer.

[20] Sng JH, Chang J, Feroze F, Rahman N, Tan W (2000). The prevalence of *BRCA1* mutations in Chinese patients with early onset breast cancer and affected relatives.. Br. J. Cancer.

[21] Patmasiriwat P, Bhothisuwan K, Sinilnikova OM, Chopin S, Methakijvaroon S (2002). Analysis of breast cancer susceptibility genes *BRCA1* and *BRCA2* in Thai familial and isolated early-onset breast and ovarian cancer.. Hum Mutat.

[22] Saxena S, Szabo CI, Chopin S, Barjhoux L, Sinilnikova O (2002). *BRCA1* and *BRCA2* in Indian breast cancer patients.. Hum. Mutat..

[23] Zhi X, Szabo C, Chopin S, Suter N, Wang QS (2002). *BRCA1* and *BRCA2* sequence variants in Chinese breast cancer families.. Hum Mutat..

[24] Balraj P, Khoo AS, Volpi L, Tan JA, Nair S (2002). Mutation analysis of the *BRCA1* gene in Malaysian breast cancer patients.. Singapore Med J..

[25] Sim X, Ali RA, Wedren S, Goh DL, Tan CS (2006). Ethnic differences in the time trend of female breast cancer incidence: Singapore, 1968–2002.. BMC Cancer.

[26] Suter NM, Ray RM, Hu YW, Lin MG, Porter P (2004). *BRCA1* and *BRCA2* mutations in women from Shanghai China.. Cancer Epidemiol. Biomarkers Prev..

[27] Abkevich V, Zharkikh A, Deffenbaugh AM, Frank D, Chen Y (2004). Analysis of missense variation in human *BRCA1* in the context of inter-specific sequence variation.. J. Med. Genet..

[28] Durocher F, Shattuck-Eidens D, McClure M, Labrie F, Skolnick MH (1996). Comparison of *BRCA1* polymorphisms, rare sequence variants and/or missense mutations in unaffected and breast/ovarian cancer populations.. Hum Mol Genet..

[29] Fackenthal JD, Sveen L, Gao Q, Kohlmeir EK, Adebamowo C (2005). Complete allelic analysis of *BRCA1* and *BRCA2* variants in young Nigerian breast cancer patients.. J Med Genet..

[30] Ng PC, Henikoff S (2003). SIFT: predicting amino acid changes that affect protein function.. Nucleic Acids Res.

